# A Double Barrier Technique with Hydrotalcites for Pb Immobilisation from Electric Arc Furnace Dust

**DOI:** 10.3390/ma12040633

**Published:** 2019-02-20

**Authors:** Angélica Lozano-Lunar, Enrique Fernández Ledesma, Álvaro Romero Esquinas, José Ramón Jiménez Romero, José María Fernández Rodríguez

**Affiliations:** 1 Department of Rural Engineering, School of Engineering Science of Belmez, Universidad de Córdoba, 14240 Córdoba, Spain; p82lolua@uco.es; 2 Department of Mechanics, School of Engineering Science of Belmez, Universidad de Córdoba, 14240 Córdoba, Spain; efledesma@uco.es; 3 Department of Inorganic Chemistry and Chemical Engineering, School of Engineering Science of Belmez, Universidad de Córdoba, 14240 Córdoba, Spain; p52roesa@uco.es

**Keywords:** electric arc furnace dust, hydrotalcite, double barrier technique, leaching behaviour, Pb immobilisation

## Abstract

A new line of mortars incorporating hydrotalcites was developed. This research article shows the results of a study of a double barrier technique (DBT) for Pb immobilisation from electric arc furnace dust (EAFD) in mortars with the addition of three different hydrotalcites (H1, H2, and H3). Electric arc furnace dust (EAFD) is a hazardous waste due to its heavy metal leachability. The aim was to obtain a mortar in which, due to its chemical composition, heavy metal leaching satisfied environmental criteria. Previously, a physical and chemical characterisation of mortar material components was carried out. The leaching behaviour of Pb from EAFD in double barrier (DB) mortars with different hydrotalcites was analysed for compressive strength to determine treatment effectiveness. DB mortars could be considered monoliths because their compressive strengths were higher than 1 MPa but exhibited a decrease due to hydrotalcite incorporation. The mortar EAFD25_H2 (with ethylenediaminetetraacetate (EDTA) in the interlayer of the hydrotalcite) showed one minor reduction in compressive strength with respect to the reference mortar because formation of Portlandite was observed, which is a characteristic of cement hydration. The conventional immobilisation mortar (EAFD25) did not achieve Pb immobilisation. However, DB mortars with dimercaptosuccinate (DMSA) in the interlayer of the hydrotalcite reduced Pb release by ~50%, from 20.29 mg kg^−1^ (EAFD25) to 9.88 mg kg^−1^ (EAFD25_H3). In addition, EAFD25_H3 included the lowest hydrotalcite content, thereby improving the immobilisation ratio. The results of this study contribute to better Pb immobilisation, thus satisfying environmental criteria.

## 1. Introduction

Human industrial activity inevitably produces waste. In the metallurgical industry, during the melting of scrap in an arc electric furnace, volatile elements vaporise and later condense [1]. Then, they are collected in bag filters and recovered as a powder called electric arc furnace dust (EAFD). These wastes are composed of heavy and transition metals such as Pb, Zn, Cd, Cr, or Cu, as well as silicates and sulphates [2]. 

EAFD is included in the European Waste Catalogue [3] as a hazardous waste due to the high heavy metal content in its chemical composition. Leaching of these heavy metals causes an adverse environmental impact that requires treatment and storage in an appropriate place. 

It is known that around 70% of EAFD is landfilled and the remaining 30% is processed for Zn recovery and other purposes [4,5,6]. However, EAFD is made up of very fine particles (0.10–200 μm) [2,7] and its handling is problematic without previous treatment. Therefore, the first action must be that EAFD disposal in hazardous waste landfills is protected from rain, to prevent contaminating leachates that could pollute surrounding areas. Because EAFD is dust, it cannot be landfilled in a natural state.

The main types of EAFD treatment technologies are pyrometallurgical, hydrometallurgical, vitrification and stabilisation/solidification (S/S) [8]. Immobilisation of hazardous waste by S/S techniques is one of the most commonly used EAFD treatment technologies and it has been pursued for many years [9]. The aim of this technology is to obtain solid blocks that are not hazardous or that are less hazardous than the original wastes using two stages that can be either consecutive or simultaneous—first, chemical stabilisation of the waste, and second, solidification. The solid blocks generated by an S/S process are checked through their mechanical behaviour and leaching behaviour in a monolithic and/or granular state. These solid blocks can be accomplished with cement as a main or partial binder [8,10,11]. 

Cement-based materials are particularly well-suited for the immobilisation of inorganic wastes due to the high pH (~12) during the cement hydration process. This favours the insolubilisation and encapsulation of metals by the formation of hydration products [12]. Nevertheless, the leaching mechanisms of certain heavy metals are still not completely defined. Authors such as Salihoglu and Pinarli [13] used lime and Portland cement as a binder in S/S of EAFD. They obtained leach values for Zn and Pb lower than those obtained in the S/S of EAFD using only lime or only Portland cement as the main binder. Cubukcuoglu and Ouki [14] reported granular leaching results with the fixing of Pb, Cd, Cr, Mo and Zn in pastes with low grade MgO as a partial replacement of Portland cement with an addition of 40% of EAFD. Issa et al. [1] observed liberation of Zn using cement as a solid matrix. Ledesma et al. [15] reduced EAFD polluting potential through S/S treatment with mortars using Portland cement, a limestone filler and siliceous natural sand. EAFD was incorporated in a ratio of 2:1 by weight (mortar−EAFD). These authors achieved a Pb reduction with the percentage of granular leaching being 99.13% with respect to the original EAFD without S/S treatment. Despite the high reduction value of Pb, the release values in the S/S mortars of EAFD exceeded the Pb limit and mortars could not be landfilled. Likewise, other authors such as Halim et al. [16], Navarro et al. [17] and Ledesma et al. [18] analysed the challenges of Pb immobilisation in cement-based materials. The highly alkaline environment in cement-based materials produced a high Pb release, because the solubility of amphoteric metals, such as Pb, varies with pH [17]. The authors concluded that the fixing mechanisms of Pb are not sufficiently known and the use of cement-based materials could be a limiting factor for Pb immobilisation [19]. A recent study carried out by Lozano-Lunar et al. [20] achieved Pb immobilization with highly alkaline cement-based materials. In their investigation, Pb release remained below the established limit by the European Council Decision 2003/33/EC [21] due to the dense matrix used in the study. Therefore, it is necessary to develop new immobilisation mortars suitable for the fixation of these types of elements. 

Other methods applied for the elimination of heavy metals were chemical precipitation into sulphurs or hydroxides, filtration, coagulation, and electrolysis [22,23,24]. Among these technologies, adsorption offers major advantages because it is more economical, highly efficient for the elimination of heavy metals, and simpler to manage. A large number of studies on the elimination of inorganic pollutants by means of adsorption technologies confirmed its utility [25,26].

Layered double hydroxides (LDH) are laminar ionic compounds that contain sheets loaded with positive charges and with exchangeable anions in the interlayers. In general, these materials are defined by the following formula [27,28] (Equation (1)):

(1) $${\left[M_{1-x}^{2+}M_{x}^{3+}{\left(\mathrm{OH}\right)}_{2}\right]}^{x+}\left[X_{x/m}^{m-}\right]\cdot {\mathrm{nH}}_{2}O^{x-}$$

where M^2+^ = Mg^2+^, Mn^2+^, Zn^2+^; M^3+^ = Al^3+^, Fe^3+^, Cr^3+^; and X^−^ = ${\mathrm{CO}}_{3}^{2-}$, Cl^−^, NO^3^^−^.

The structure of LDH, as well as their ability for anionic exchange, make them suitable for many applications [29]. Adsorbents are selected depending on their properties, which can be modified with chelating agents such as ethylenediaminetetraacetate (EDTA) [30,31], nitrilotriacetate (NTA) [32,33], diethylenetriamine pentaacetate (DTPA) and dimercaptosuccinate (DMSA) [25]. The interlayer modification in hydrotalcites by chelating agents benefits metal adsorption [25,34,35]. 

The literature review showed that Cu was best captured using a hydrotalcite with EDTA in the interlayer, which also produced good data for Pb [31]. The best reported results for the immobilisation of Pb used hydrotalcites with DMSA in the interlayer [25].

LDH can also be used as active components of cement and mortars [36], due to their compatibility with cementitious materials [37].

The present paper describes a study of a double barrier technique (DBT) for the immobilisation of Pb from EAFD in cement-based mortars with the addition of organohydrotalcites containing EDTA or DMSA in the interlayer. These immobilisation mortars were called double barrier (DB) mortars. The hydrotalcite chemically stabilised the Pb of EAFD, while the solidification process occurred with cement-based mortars. Additionally, a hydrotalcite with a carbonate in the interlayer was used. It is very important to highlight the high amount of EAFD that this study attempted to immobilise—around 25% in weight replacing a siliceous filler. The degree of immobilisation was studied by means of the evolution of the compressive strength and leaching behaviour.

## 2. Materials and Methods 

### 2.1. Characterisation of Mortar Component Materials

For mortar production, cement CEMI/52.5 R (CEM) made by Cementos Cosmos S.A. (Córdoba, Spain) in accordance with JOINS IN 80303-1 and JOINS IN 197-1 [38] was used, the composition of which was provided by the manufacturer. A siliceous filler (SF) supplied by Lorda and Roig S.A. (Barcelona, Spain) and siliceous natural sand (NS) obtained from Áridos Álvarez (Córdoba, Spain) were used. Pb was obtained from the waste of steel mill electric arc furnace dust (EAFD) supplied by the Olaberría steel mill in Guipúzcoa, Spain. Sampling was carried out in accordance with UNE-EN 14899:2007 [38] from the fume extraction duct of steelworks. The composition of SF, NS, and EAFD expressed in oxides was determined by energy dispersive analysis of X-rays (EDAX) with scanning electronic microscopy (SEM) using JSM-6300 equipment (Jeol, Tokyo, Japan) at a potential of acceleration of 20 kV and a distance of 15 mm.

Fourier transform infrared (FTIR) spectroscopy of EAFD was accomplished with a Bruker Tensor 27-Hyperion 2000 FT-MIR spectrophotometer (Billerica, MA, USA). OPUS 6.5 software (Bruker) was used to collect the transmission spectra.

Grain-size distributions of CEM, SF, and EAFD were measured in a Malvern Mastersizer S analyser (Malvern, UK), and the particle size distribution of NS was determined in accordance with UNE-EN 933-1:2012 [38].

The main crystalline phases were analysed by X-ray diffraction patterns (XRD) using a Bruker D8 Discover A25 instrument with Cu−Kα radiation. All diffraction patterns were obtained by scanning the goniometer from 5° to 70° (2θ) at a speed of 0.15° s^−1^ for CEM, SF, NS and hydrotalcites, and for EAFD, by scanning the goniometer from 5° to 70° (2θ) at a speed of 0.02° s^−1^. 

The specific surface area of EAFD and SF was measured using the Brunauer–Emmett–Teller (BET) method by the absorption of N_2_ with a Micromeritics ASAP 2010 instrument (Norcross, GA, USA). The specific surface area of CEM was provided by Cementos Cosmos S.A. SEM images of EAFD were obtained with JSM-6300. The specific gravity of EAFD was calculated in accordance with UNE-EN 80103:2013 [38]. The specific gravity of SF and CEM was provided by the manufacturers. Furthermore, the NS specific gravity and water absorption were calculated in accordance with UNE-EN 1097-6:2014 [38].

Three different hydrotalcites called H1, H2, and H3 were used. The first one (H1) was a hydrotalcite with Zn and Al in the sheet and ${\mathrm{CO}}_{3}^{2-}$ in the interlayer, synthesised by a co-precipitation method at a fixed pH of 10. The synthesis procedure was as follows: A watery solution containing Zn(NO_3_)_2_·6H_2_O (0.75 M) and Al(NO_3_)_3_·9H_2_O (0.25 M) was added drop wise to a stirred solution of NaOH (2 M) and 0.2 M of ${\mathrm{CO}}_{3}^{2-}$ anion. The resulting gel was hydrothermally treated at 80 °C for 24 h to improve the crystallinity degree of the hydrotalcite. Then, the gel was filtered and washed repeatedly with deionised water. Lastly, it was dried at 60 °C for 24 h [39]. 

H2 and H3 were synthesised in a laboratory as follows: First, a hydrotalcite with Zn and Al in the layers and NO_3_^−^ in the interlayer was synthesised ([Zn_2_Al(OH)_6_]NO_3_·nH_2_O). This synthesis was realised by a precipitation method at room temperature [40] in accordance with Perez et al. [31]. 

Next, to introduce EDTA in the interlayer, an ion exchange method under a N_2_ flow at 75 °C in accordance with Perez et al. [31] was used. From nitrate hydrotalcite synthesis, a nitrate hydrotalcite suspension (150 mL) was added to 0.015 mol of Na_2_H_2_Y (where Y was the EDTA^2^^−^ anion). pH was kept at 5.5, at which level the ligand exists mainly as [H_2_Y]^2^^−^, thus allowing intercalation by anion-exchange with LDH as follows (Equation (2)):

(2) $$2\left[{\mathrm{Zn}}_{2}\mathrm{Al}{\left(\mathrm{OH}\right)}_{6}\right]{\mathrm{NO}}_{3}+{\left[H_{2}Y\right]}^{2-}\to {\left[{\mathrm{Zn}}_{2}\mathrm{Al}{\left(\mathrm{OH}\right)}_{6}\right]}_{2}\left[H_{2}Y\right]+2{\mathrm{NO}}_{3}^{-}.$$

The final gel, ZnAl-EDTA (H2), was centrifuged, washed with deionised water, and dried at 60 °C for 24 h.

Finally, to introduce DMSA into the interlayer, 0.015 mol of DMSA was dropped into 150 mL of a nitrate hydrotalcite suspension under a N_2_ stream at 75 °C in accordance with Pavlovic et al. [25]. pH was maintained at 5.5 throughout this synthetic procedure because the aqueous dissociation constants of DMSA were pK_1_ = 2.71, pK_2_ = 3.43, pK_3_ = 9.65, and pK_4_ = 12.05 [41], which was suitable for the anion-exchange reaction. Also, pH 5.5 guaranteed minimal dissolution of atmospheric CO_2_. The final product, ZnAl-DMSA (H3), was separated by centrifugation, washed with deionised water, and dried at 60 °C for 24 h.

### 2.2. Leaching and pH Dependence of EAFD

The influence of pH on leaching of Pb from EAFD was analysed in accordance with UNE-EN 14429:2015 [38]. Eight samples were studied at different pH values in the range of pH 4–12. Acid or base was added as needed to obtain the desired pH values. As soon as the addition had been completed, demineralised water was added to obtain a liquid/solid ratio (L/S) of 10. After 48 h, eluates were extracted at the different pH values and analysed by ICP-MS using a Perkin Elmer ELAN DRC-e spectrometer (Waltham, MA, USA).

A compliance test of EAFD, in order to evaluate Pb leaching behaviour and to analyse its potential hazard, as well as its environmental classification, was performed (UNE-EN 12457-4:2003) [38]. Dried EAFD (90 g) was mixed with 900 g of demineralized H_2_O (<0.5 mS cm^−1^) to give an L/S ratio of 10. After 24 h, eluates were extracted and analysed by ICP-MS. The release results were compared with the limit established for Pb leaching in the European Council Decision 2003/33/EC [21].

### 2.3. Immobilisation Mortar Dosage 

In the first stage, a total of five mixtures were made. From a reference mortar (RM) composed of CEM, NS, and SF, four conventional immobilisation mortars were manufactured by substituting 25, 50, 75, and 100% of SF by weight with EAFD. These were called EAFD25, EAFD50, EAFD75, and EAFD100, respectively.

In the second stage, DB mortars were obtained by the addition of three different hydrotalcites (H1, H2, and H3) to EAFD25 to give EAFD25_H1, EAFD25_H2, and EAFD25_H3, respectively. The conventional immobilisation mortar was chosen due to its better mechanical behaviour under compression compared with the rest. The properties of the eight mortars are shown in Table 1.

The quantity of incorporated hydrotalcite was calculated in accordance with the Pb adsorption capacity of each of them, which was reported by Perez et al. [31] and Pavlovic et al. [25] for H2 and H3, respectively (Table 1). The quantity of added water was calculated in an experimental procedure in which a plastic consistency (C) of 175 ± 10 mm in a flow table was obtained in accordance with UNE-EN 1015-3:2000 [38]. This procedure consisted of adding water experimentally to the mixture and checking the consistency on the flow table. If the mortar consistency had a value of 175 ± 10 mm, the added water was validated. When the values did not meet the criteria, the water amount was changed, checking the consistency value again. 

### 2.4. Mixing Procedure

The mixing procedure used for RM, conventional immobilisation mortars, and DB mortars was the same. Water, cement, filler/EAFD, sand, and hydrotalcite were added sequentially over a period of 3 min and then mixed for 2 min. From each of the mixtures, 10 cylindrical mortar samples were taken with diameters of 40 mm and heights of 80 mm.

### 2.5. Compressive Strength and Leaching Behaviour of Immobilisation Mortars 

To verify Pb immobilisation in conventional and DB mortars and its effect on mechanical stability, compressive strength and leaching properties were measured in mortar samples at 28 days. Curing conditions were a temperature of 20 °C ± 2 °C and a relative humidity of 95% ± 5%. 

The compressive strength of the hardened mortars was evaluated (XP X31-212:2011) [42]. All immobilisation mortars must exceed 1 MPa of compressive strength to be considered as immobilisation monoliths.

The test UNE-EN 12457-4:2003 [38] was used with hardened mortars. A preliminary sample preparation was carried out. Mortar samples at 28 days were crushed and sieved by a 10 mm sieve before the compliance test. After 24 h, eluates were extracted and analysed by ICP-MS. The results were compared with the limit established for Pb by the European Council Decision 2003/33/EC [21].

## 3. Results and Discussion

### 3.1. Characterisation of Mortar Component Materials

**Figure 1:**
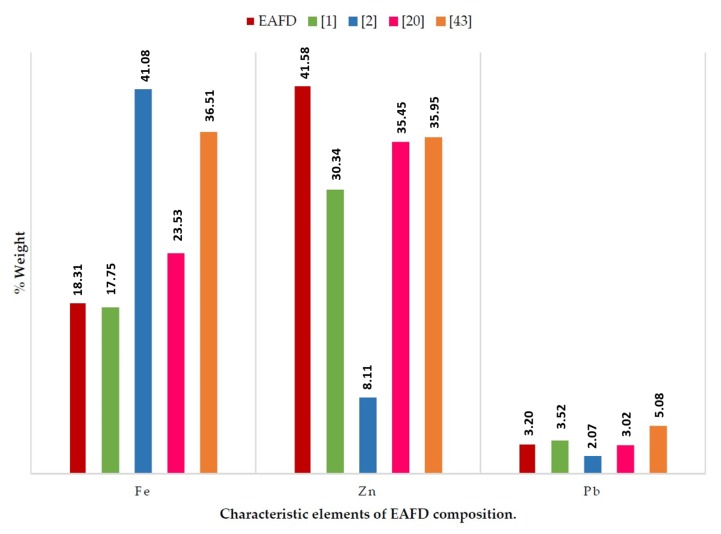
Chemical composition of EAFD and comparison with previous reports.

Table 2 and Table 3 show the composition of the mortar materials. SF and NS have a siliceous character. The major elements in EAFD were Zn and Fe at 41.58% and 18.31%, respectively. The amount of Pb was of the same order as that found in the information contributed by other authors (Figure 1) [1,2,20,43]. 

**Figure 2:**
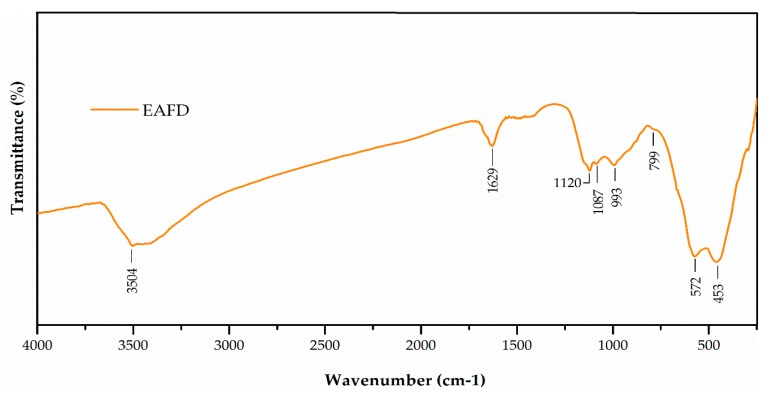
Fourier-transform infrared (FT-IR) spectrum of EAFD.

The Fourier-transform infrared (FT-IR) spectrum of EAFD (Figure 2) shows bands at 3504 and 1629 cm^−1^, which were characteristic of stretching and bending vibrations, respectively, of the OH^−^ groups [44]. The bands at 1170 cm^−1^ and 1028 cm^−1^ were related to the vibration of asymmetric Si–O–Si stretching, whereas the band at 799 cm^−1^ corresponded to the symmetrical Si–O–Si stretching, and the last peak at 453 cm^−1^ was related to the O–Si–O bending vibration. The band located at 577 cm^−1^ was related to the bending vibration O–Al–O [45]. The band located at 1087 cm^−1^ may have been indicative of the presence of sulphates [46]. The bands located at 1425 and 877 cm^−1^ may have been indicative of the presence of carbonates [46]. These results agree with those presented in Table 3.

**Figure 3:**
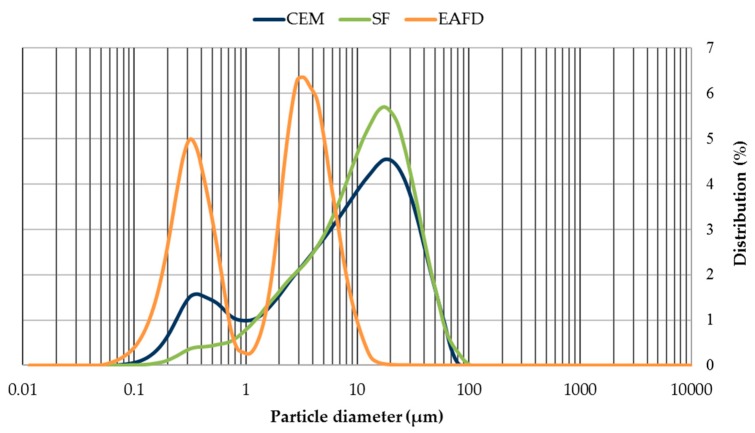
Grain-size distribution of cement (CEM), siliceous filler (SF) and EAFD.

**Figure 4:**
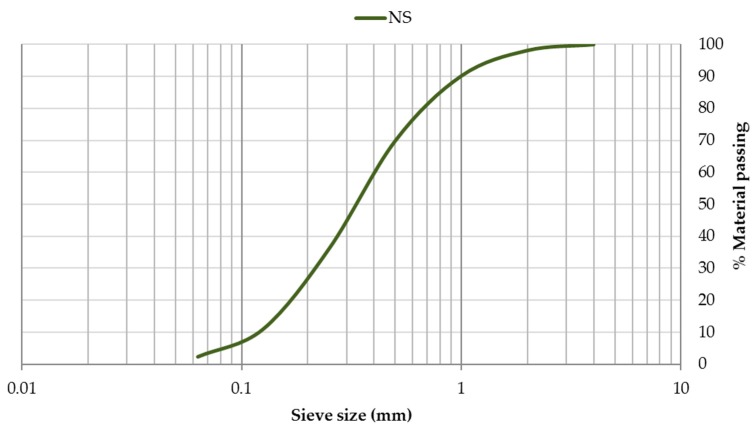
Particle size distribution of natural sand (NS).

The grain-size distribution of CEM, SF, and EAFD is represented by Figure 3. CEM and SF particles were observed to be homogeneous with sizes between 0.06–100 µm. Both materials included a large percentage of particles around 20 µm, which was the main percentage for SF. The grain size of EAFD was from 0.05 µm to 20 µm, which was consistent with the findings of Sofilic et al. [2] and Lenz and Martins [7]. The grain-size distribution was bimodal, showing two maxima around 0.3 µm and 3 µm. This bimodal distribution agreed with that obtained by Oustadakis et al. [5]. In addition, the particle size distribution of NS at mass (Figure 4) was shown, which had a maximum size of 2 mm (UNE-EN 933-2:1996) [38].

**Figure 5:**
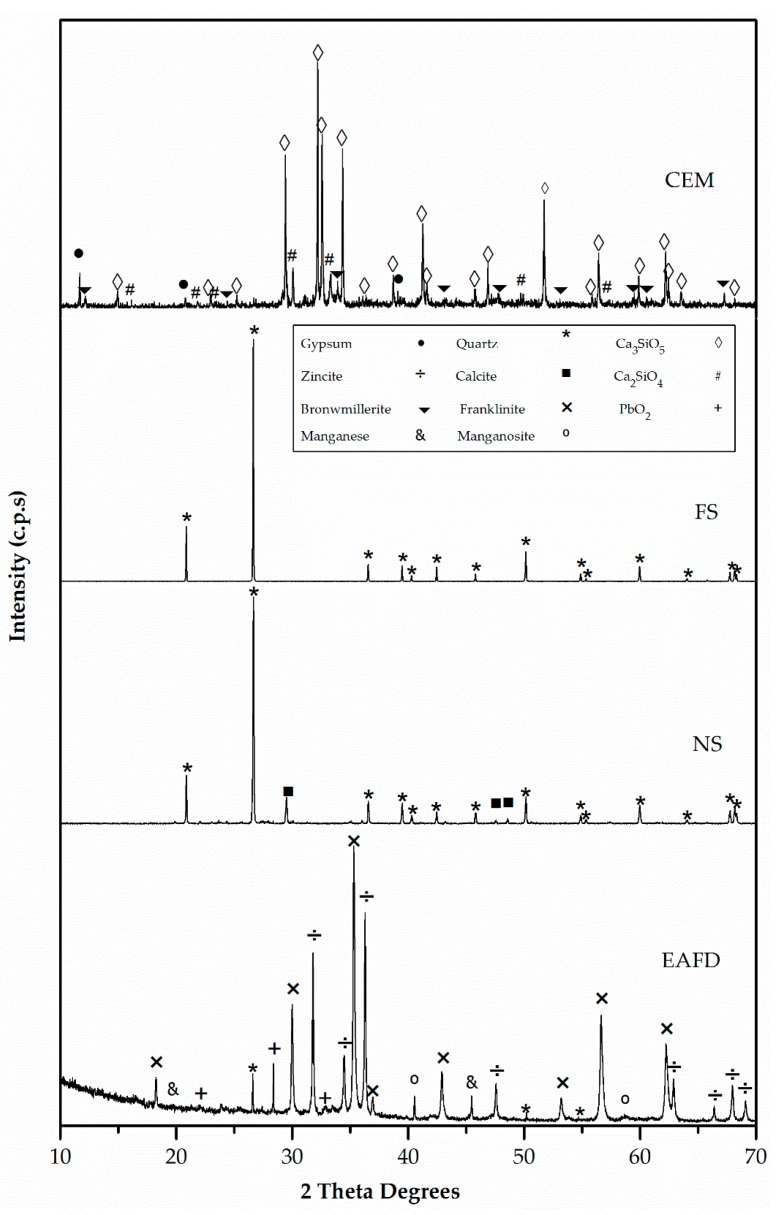
X-ray diffraction (XRD) patterns of cement (CEM), siliceous filler (SF), natural sand (NS) and electric arc furnace dust (EAFD).

**Figure 6:**
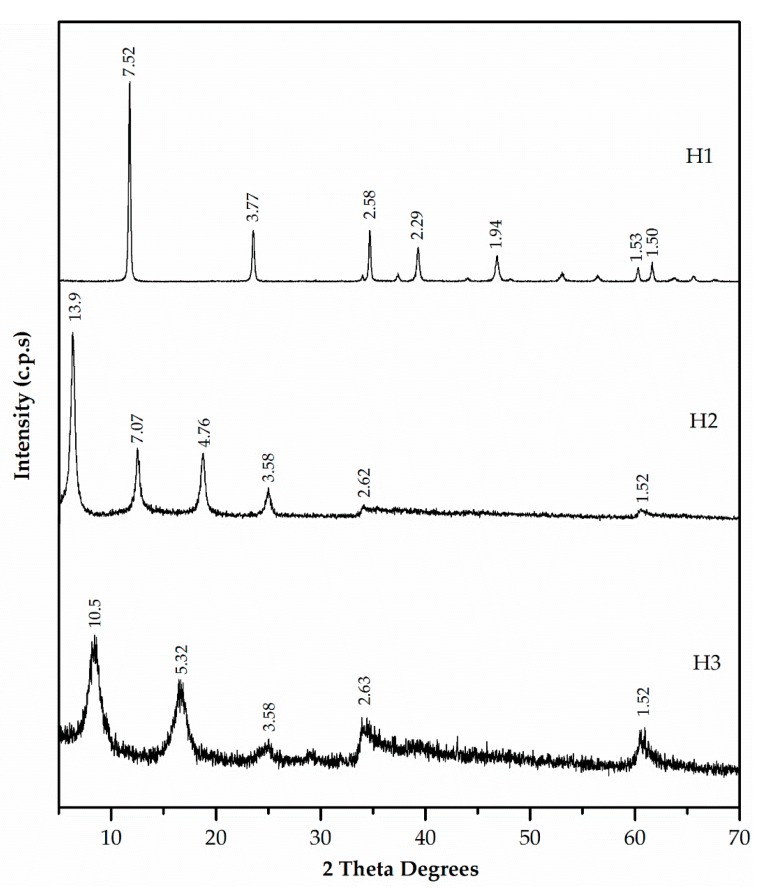
XRD patterns of hydrotalcites H1, H2 and H3.

Figure 5 and Figure 6 exhibit the XRD patterns of mortar component materials. CEM consisted essentially of tricalcium silicate (Ca_3_SiO_5_) (42-0551), dicalcium silicate (Ca_2_SiO_4_) (24-0034), and, to a lesser extent, gypsum (CaSO_4_·H_2_O) (33-0311) [47]. The only observed phase in FS was silica oxide (SiO_2_) (33-1161) [47]. The major phase in NS was quartz (SiO_2_) (33-1161) with a small amount of calcium carbonate (CaCO_3_) (05-0586) [47]. EAFD waste was a polymetallic mixture of different oxide components [18]. The main phases were franklinite (ZnFe_2_O_4_) and zincite (ZnO). In addition, metallic Mn, such as MnO, quartz (SiO_2_), and PbO_2_ were also detected [20]. 

The presence of franklinite and zincite was related to the observed Zn content, which was higher than the stoichiometric ratio of Zn and Fe in the spinel [18]. Although EAFD composition depended on the scrap metal [2], the XRD results of EAFD were in agreement with those obtained by Suetens et al. [48] and Lozano-Lunar et al. [20].

Figure 6 presents the XRD patterns of the three synthesised hydrotalcites. These patterns were characteristic of a layered phase. H1, H2, and H3 samples exhibited values of d(003) = 7.52, 13.9, and 10.5 Å, respectively, and values of d(006) = 3.77, 7.07, and 5.32 Å, respectively. The increase in basal spacing from d = 8.8 Å (ZnAl-NO_3_) to d = 13.9 Å and d = 10.5 Å in H2 and H3, respectively, indicated EDTA and DMSA intercalation in ZnAl-NO_3_ (not included in Figure 6). 

**Figure 7:**
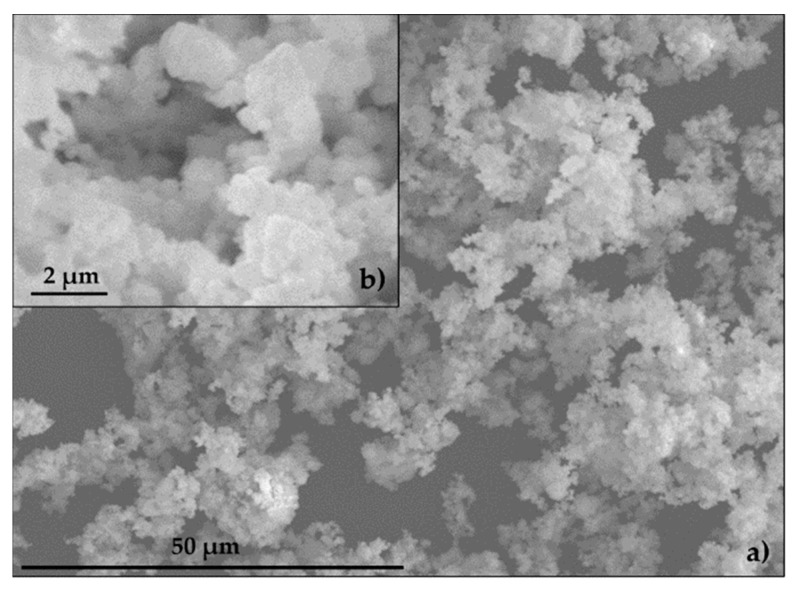
Scanning electron microscopy (SEM) of EAFD: (**a**) 50 μm; (**b**) 2 μm.

The specific surface of EAFD was 3.70 m^2^ g^−1^. The specific surfaces for SF and CEM were provided by the manufacturer and registered values of 0.25 m^2^ g^−1^ and 0.35 m^2^ g^−1^, respectively. Figure 7 shows a group of small particles (~1 µm) combining to form particles of 10–15 µm in size, consistent with the findings of other authors [7,49,50,51]. The EAFD specific gravity was 3.85 g cm^−3^, which was similar to values obtained by other authors [13,52,53]. The specific gravity of SF and CEM, provided by the manufacturer, was 2.60 g cm^−3^ and 3.14 g cm^−3^, respectively. The NS specific gravity and water absorption were 2.63 g cm^−3^ and 0.23%, respectively.

### 3.2. Leaching and pH Dependence of EAFD

**Figure 8:**
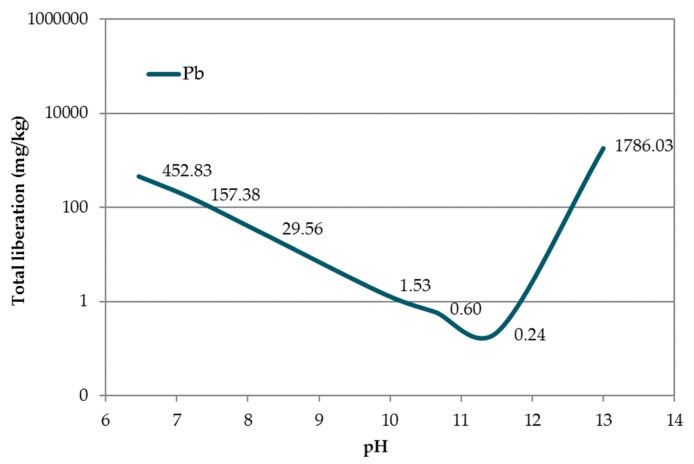
pH dependence of Pb contained in EAFD.

The pH dependence of Pb liberation from acidic (pH 6.5) to basic (pH 13) pH in accordance with UNE-EN 14429:2015 [38] is shown in Figure 8. The minimum of the curve (corresponding to a leaching value of 0.60 mg kg^−1^) appears at pH 11.20, which was higher than that reported by van der Sloot and Dijkstra [54], likely due to the different matrices in which the Pb was located. 

In Table 4, the leaching results of Pb from the EAFD sample are shown, as well as the pH value, temperature (°C), and conductivity (mS cm^−1^) of the eluate extracted (UNE-EN 12457-4:2003) [38]. In consideration of the European Council Decision 2003/33/EC [21] in relation to Pb’s liberation, EAFD was classified as ‘not hazardous’ (6.30 mg of Pb liberated per kg of dry matter). The total amount of Pb liberated was 0.02% of the initial quantity (32,000 mg kg^−1^) in EAFD.

### 3.3. Compressive Strength at 28 Days

**Figure 9:**
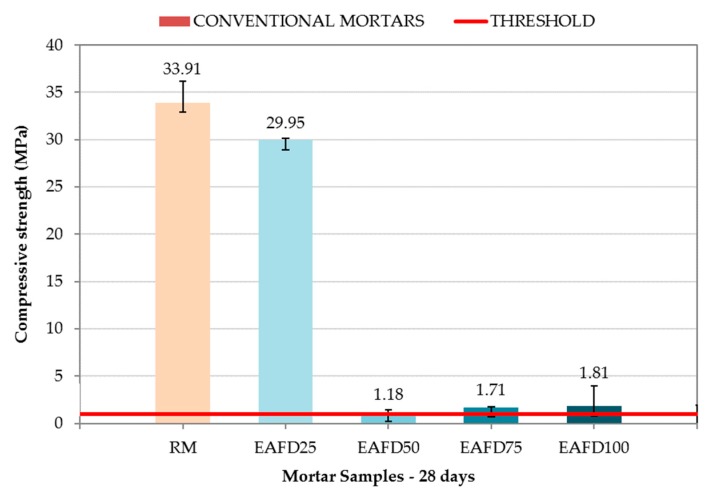
Compressive strength of the reference mortar (RM) and conventional immobilisation mortars.

Figure 9 shows the compressive strength of RM and conventional immobilisation mortars at 28 days. In addition, the compressive strength that mortars must achieve in order to be considered as immobilisation monoliths (1 MPa) is indicated by a red line.

RM exhibited a compressive strength of 33.91 MPa, but resulted in a loss of the same magnitude (29.95 MPa) with the incorporation of 25% by mass of EAFD (EAFD25). The conventional immobilisation mortars EAFD50, EAFD75, and EAFD100 exhibited a sudden drop in compressive strength with values of 1.18, 1.71, and 1.81, respectively, with the increase in heavy metal content. Heavy metals influence cement hydration and, therefore, the acquisition of compressive strength [15,18,55].

The compressive strength results were in agreement with those observed by Belebchouche et al. [56] and Chaabane et al. [10], although the percent drop in compressive strength for the same amount of waste as the total mixture in the present study was lower. Those authors observed a sudden drop in compressive strength for small amounts of waste, and a slight growth for a certain amount of added waste, followed by a slight decrease later. Despite the sudden loss of compressive strength, all of the studied conventional immobilisation mortars were still considered monoliths because they maintained a compressive strength over the 1 MPa limit.

The mortar EAFD25 was selected for the next stage because of its favourable mechanical behaviour. Three hydrotalcites were added to this conventional immobilisation mortar to produce DB mortars.

**Figure 10:**
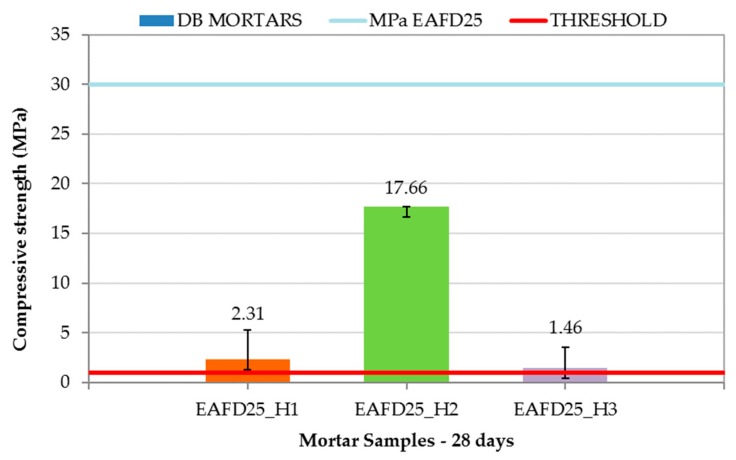
Compressive strength of Double Barrier (DB) mortars.

The DB mortar results are shown in Figure 10, along with the compressive strength limit of 1 MPa (red line) and that of EAFD25 (29.95 MPa). The addition of hydrotalcites to the conventional immobilisation mortar caused a decrease in compressive strength with values of 2.31, 17.66, and 1.46 MPa for EAFD25_H1, EAFD25_H2, and EAFD25_H3, respectively. A similar behaviour was observed by Yang et al. [36], who noticed a decrease in compressive strength when introducing hydrotalcites as a cement substitute. Cao et al. [57] also recorded a decreasing tendency of compressive strength in mixes of cement with hydrotalcite at 28 days. These authors attributed the compressive strength loss to the formation of a protective cover over cement particles that decreased the hydration reaction of the cement. 

Although DB mortars suffered a decrease in compressive strength compared to EAFD25, they all exceeded the 1 MPa requirement for consideration as a monolith. The high compressive strength obtained by the EAFD25_H2 mortar (17.66 MPa), whose hydrotalcite incorporated EDTA in the interlayer, is worth emphasising.

**Figure 11:**
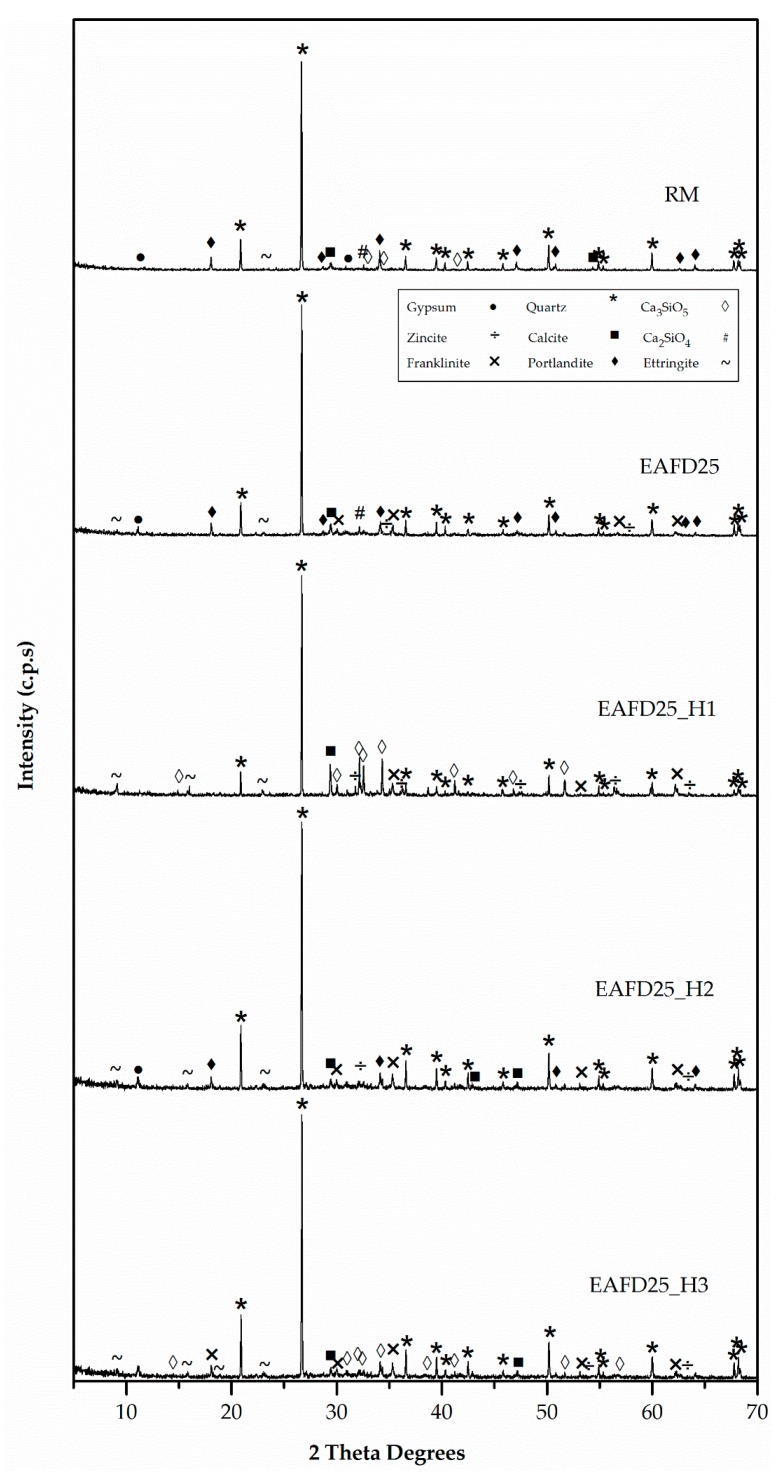
XRD patterns of DB mortars.

These facts were in accordance with Figure 11 (XRD patterns), in which the compressive strength behaviour of mortars could be attributed to the formation of the Portlandite phase [15,18,20], since this phase only appeared in mortars with good mechanical properties (RM, EAFD25, and EAFD25_H2) and was not present in EAFD25_H1 or EAFD25_H3. These results were in agreement with those in Ledesma et al. [15,18] and Lozano-Lunar et al. [20] who showed that Portlandite (Ca(OH)_2_) was formed due to the extension of the Portland cement reaction. Hydrotalcite, due to its acid-base characteristics, to a greater or lesser extent, inhibits the hydration reaction of cement, which translates into a minor amount or absence of Portlandite (Ca(OH)_2_).

In addition, this agrees with the absence of Ca_2_SiO_4_ and Ca_3_SiO_5_ phases in the EAFD25 and EAFD25_H2 mortars and their presence in the EAFD25_H1 and EAFD25_H3 mortars. 

### 3.4. Leaching Behaviour of Immobilisation and DB Mortars

Table 4 shows the Pb leaching results of RM and the conventional mortar EAFD25 and of DB mortars EAFD25_H1, EAFD25_H2, and EAFD25_H3, as well as the pH, temperature (°C), and conductivity (mS cm^−1^) of the eluates extracted (UNE-EN 12457-4: 2003) [38].

No elements were leached in RM over the ‘inert’ limit (European Council Decision 2003/33/EC) [21]. The conventional mortar EAFD25 did not meet the Pb immobilisation requirement, because it produced leaching concentrations above the ‘not hazardous’ limit. This Pb concentration (20.29 mg kg^−1^) was even higher than the leached concentration of EAFD. This phenomenon was explained by the pH increase during cement hydration due to the presence of OH^−^ ions. The natural pH of EAFD was 8.73. Its incorporation into the cementitious matrix in EAFD25 resulted in a pH value of 12.54. This pH difference has been postulated as a limiting factor for the use of cement-based mortars in S/S processes [19]. In Figure 8, a highly alkaline medium that is detrimental to Pb immobilisation was observed, since it is an amphoteric element and its leaching is highly pH dependent. This phenomenon was reported by Navarro et al. [17], who stated that ordinary Portland cement (OPC) was not suitable for precipitation of some metals, including Pb. It is for this reason that a new line of research arose using DB mortars, consisting of hydrotalcites added to conventional mortars, to immobilize the Pb contained in EAFD.

In accordance with the European Council Decision 2003/33/EC [21] regarding Pb release, mortars EAFD25_H1 and EAFD25_H2 were classified as ‘hazardous’ (Pb released > 10 mg kg^−1^). The EAFD25_H3 mortar was classified as ‘not hazardous’ (Pb released < 10 mg kg^−1^). In all DB mortars, a decrease in the released Pb amount with respect to the conventional mortar was observed, very close to the limit of 10 mg kg^−1^. An approximately 50% reduction in the Pb release was achieved, from 20.29 mg kg^−1^ (EAFD25) to 9.88 mg kg^−1^ (EAFD25_H3), coinciding with the minimum pH shown in Figure 8.

Hydrotalcites added into the mortars served, on one hand, as a pH modulator of the medium, and, on the other hand, depending on their composition, as a Pb capture agent. Specifically, EAFD25_H2 and EAFD25_H3, which possess this double function, were those that achieved the greatest Pb immobilisation. EAFD25_H3 produced the highest immobilisation; even though its compressive strength was inferior to EAFD25_H2, it complied fully with the mechanical requirements established in XP X31-212:2011 [42].

## 5. Conclusions

The present paper reveals the results of a preliminary study of a double barrier technique (DBT) for Pb immobilisation using EAFD in cement-based mortars and the addition of three hydrotalcites. EAFD was a mixture of metal oxides, the majority of which were Zn, Fe, and Pb oxides.

Conventional immobilisation mortar EAFD25 did not satisfy the Pb immobilisation requirement, since the highly alkaline medium was detrimental to immobilisation of said element. The percentage of Pb leaching was 0.63%.

A new line of mortars incorporating hydrotalcites was used. DB mortars exhibited decreased compressive strength due to the incorporation of hydrotalcites. All DB mortars were considered as monoliths because they exceeded the 1 MPa compressive strength limit. The mortar with the smallest compressive strength reduction was EAFD25_H2, which incorporated a hydrotalcite with EDTA in the intersheet and in which the Portlandite phase, characteristic of the hydration of cement, was formed. 

EAFD25_H1 and EAFD25_H2 mortars were considered ‘hazardous’, whereas EAFD25_H3 could be classified as ‘not hazardous’. In all DB mortars, a clear decrease in the amount of Pb released relative to the conventional mortar was observed. A reduction of around 50% Pb released was detected, from 20.29 mg kg^−1^ (EAFD25) to 9.88 mg kg^−1^ (EAFD25_H3), coinciding with the minimum observed in the pH dependence test. In addition, EAFD25_H3 had the lowest hydrotalcite content, improving its immobilisation ratio (i.e., a lower amount of hydrotalcite produced higher immobilisation).

The DBT based on the addition of hydrotalcites to cement-based mortars developed in the present preliminary study improved the immobilisation of Pb by 50%.

## Figures and Tables

**Table 1:** Immobilisation mortar dosage.

Mortars’ Composition		CEM (g)	NS (g)	SF (g)	EAFD (g)	H (g)	%W	C (mm)
**First Stage**								
Reference Mortar	RM	1600	800	1600	0	0	26.25	183.00
Conventional Immobilisation Mortars	EAFD25	1600	800	1200	400	0	25.97	181.25
	EAFD50	1600	800	800	800	0	24.32	171.50
	EAFD75	1600	800	400	1200	0	25.97	179.75
	EAFD100	1600	800	0	1600	0	25.00	165.00
**Second Stage**								
DB mortars	EAFD25_H1	1600	800	1200	400	63	25.97	177.30
	EAFD25_H2	1600	800	1200	400	63	25.97	177.60
	EAFD25_H3	1600	800	1200	400	34	25.97	179.50

**Table 2:** Composition of mortar component materials.

Materials	Fe_2_O_3_	CaO	SiO_2_	SO_3_	K_2_O	MgO	Al_2_O_3_
CEM ^1^	2.59	64.58	20.16	3.46	1.00	0.98	4.52
SF ^2^	-	-	100.00	-	-	-	-
NS ^2^	6.80	4.73	72.14	-	2.53	0.70	10.31

^1^ Data provided by the manufacturer. There is an unidentified remainder composition (2.71%); ^2^ Composition determined by energy dispersive analysis of X-rays (EDAX).

**Table 3:** Chemical composition of electric arc furnace dust (EAFD).

**Element**	C	O	Mg	Al	Si	S	Cl	K	Ca	Ti	Cr	Mn	Fe	Zn	Pb
**% Weight**	6.53	16.18	0.91	0.37	1.21	0.79	5.08	1.30	2.20	-	-	2.35	18.31	41.58	3.20

**Table 4:** Release of leached Pb in EAFD (mg kg^−1 3^), a conventional immobilisation mortar and DB mortars. pH, T^a^ (°C) and conductivity (mS cm^−1^).

Leaching of Pb	EAFD	RM	EAFD25	EAFD25_H1	EAFD25_H2	EAFD25_H3
Pb	6.30 ^NH^	0.00 ^I^	20.29 ^H^	13.17 ^H^	11.33 ^H^	9.88 ^NH^
pH	8.73	12.52	12.54	11.93	12.12	11.81
T^a^ (°C)	29.5	24.35	24.35	25.7	25.9	26.2
C (mS cm^−^^1^)	17.29	9.62	10.10	6.86	9.36	5.90

^3^ mg Pb kg^−1^ of EAFD or crushed mortar. Limit established for Pb in the European Council Decision 2003/33/EC [21] (mg kg^−1^ dry matter): Inert (I) 0.5; Not Hazardous (NH) 10; Hazardous (H) 50.

## References

[1] Issa H., Korac M., Gavrilovski M., Kamberovic Z. (2013). Possibility of carbon steel EAFD solidification/stabilization in concrete. Metal. Int..

[2] Sofilic T., Rastovcan-Mioc A., Cerjan-Stefanovic S., Novosel-Radovic V., Jenko M. (2004). Characterization of steel mill electric-arc furnace dust. J. Hazard. Mater..

[3] (2002). European Waste Catalog. https://www.boe.es/boe/dias/1999/01/08/pdfs/A00570-00580.pdf.

[4] Maslehuddin M., Awan F.R., Shameem M., Ibrahim M., Ali M.R. (2011). Effect of electric arc furnace dust on the properties of OPC and blended cement concretes. Constr. Build. Mater..

[5] Oustadakis P., Tsakiridis P.E., Katsiapi A., Agatzini-Leonardou S. (2010). Hydrometallurgical process for zinc recovery from electric arc furnace dust (EAFD): Part I: Characterization and leaching by diluted sulphuric acid. J. Hazard. Mater..

[6] Yoo J.M., Kim B.S., Lee J.C., Kim M.S., Nam C.W. (2005). Kinetics of the volatilization removal of lead in electric arc furnace dust. Mater. Trans..

[7] Lenz D.M., Martins F.B. (2007). Lead and zinc selective precipitation from leach electric arc furnace dust solutions. Matéria (Rio J.).

[8] Conner J.R., Hoeffner S.L. (1998). The History of Stabilization/Solidification Technology. Crit. Rev. Environ. Sci. Technol..

[9] Chen Q.Y., Tyrer M., Hills C.D., Yang X.M., Carey P. (2009). Immobilisation of heavy metal in cement-based solidification/stabilisation: A review. Waste Manag..

[10] Chaabane L., Moussaceb K., Ait-Mokhtar A. (2017). Factors affecting the leaching of heavy metals (Ni^+2^, Pb^+2^, Cr^+3^) contained in sludge waste stabilization/solidification by hydraulic benders, Part I: Water/cement and waste/cement ratio in S/S mortars. Environ. Prog. Sustain. Energy.

[11] Katsioti M., Katsiotis N., Rouni G., Bakirtzis D., Loizidou M. (2008). The effect of bentonite/cement mortar for the stabilization/solidification of sewage sludge containing heavy metals. Cem. Concr. Comp..

[12] Hills C.D., Sollars C.J., Perry R. (1994). A calorimetric and microstructural study of solidifiekd toxic wastes—Part 1: A classification of OPC waste interference effects. Waste Manag..

[13] Salihoglu G., Pinarli V. (2008). Steel foundry electric arc furnace dust management: Stabilization by using lime and Portland cement. J. Hazard. Mater..

[14] Cubukcuoglu B., Ouki S.K. (2012). Solidification/stabilisation of electric arc furnace waste using low grade MgO. Chemosphere.

[15] Ledesma E.F., Lozano-Lunar A., Ayuso J., Galvín A.P., Fernández J.M., Jiménez J.R. (2018). The role of pH on leaching of heavy metals and chlorides from electric arc furnace dust in cement-based mortars. Constr. Build. Mater..

[16] Halim C.E., Scott J.A., Amal R., Short S.A., Beydoun D., Low G., Cattle J. (2005). Evaluating the applicability of regulatory leaching tests for assessing the hazards of Pb-contaminated soils. J. Hazard. Mater..

[17] Navarro A., Cardellach E., Corbella M. (2011). Immobilization of Cu, Pb and Zn in mine-contaminated soils using reactive materials. J. Hazard. Mater..

[18] Ledesma E.F., Jimenez J.R., Ayuso J., Fernandez J.M., de Brito J. (2017). Experimental study of the mechanical stabilization of electric arc furnace dust using fluid cement mortars. J. Hazard. Mater..

[19] De Angelis G., Medici F., Montereali M.R., Pietrelli L. (2002). Reuse of residues arising from lead batteries recycle: a feasibility study. Waste Manag..

[20] Lozano-Lunar A., Raposeiro da Silva P., de Brito J., Fernández J.M., Jiménez J.R. (2019). Safe use of electric arc furnace dust as secondary raw material in self-compacting mortars production. J. Clean. Prod..

[21] (2003). European Council Decision for the Acceptance of Waste at Landfills. http://eur-lex.europa.eu/LexUriServ/LexUriServ.do?uri=OJ:L:2003:011:0027:0049:EN:PDF.

[22] Ter Heijne A., Liu F., van der Weijden R., Weijma J., Buisman C.J.N., Hamelers H.V.M. (2010). Copper Recovery Combined with Electricity Production in a Microbial Fuel Cell. Environ. Sci. Technol..

[23] Hashim M.A., Mukhopadhyay S., Sahu J.N., Sengupta B. (2011). Remediation technologies for heavy metal contaminated groundwater. J. Environ. Manag..

[24] Gonzalez M.A., Trocoli R., Pavlovic I., Barriga C., La Mantia F. (2016). Capturing Cd(II) and Pb(II) from contaminated water sources by electro-deposition on hydrotalcite-like compounds. Phys. Chem. Chem. Phys..

[25] Pavlovic I., Perez M.R., Barriga C., Ulibarri M.A. (2009). Adsorption of Cu^2+^, Cd^2+^ and Pb^2+^ ions by layered double hydroxides intercalated with the chelating agents diethylenetriaminepentaacetate and meso-2,3-dimercaptosuccinate. Appl. Clay Sci..

[26] Gonzalez M.A., Pavlovic I., Rojas-Delgado R., Barriga C. (2014). Removal of Cu^2+^, Pb^2+^ and Cd^2+^ by layered double hydroxide-humate hybrid. Sorbate and sorbent comparative studies. Chem. Eng. J..

[27] Forano C., Hibino T., Leroux F., Taviot-Guého C., Faïza Bergaya B.K.G.T.a.G.L. (2006). Chapter 13.1 Layered Double Hydroxides. Developments in Clay Science.

[28] Cavani F., Trifirò F., Vaccari A. (1991). Hydrotalcite-type anionic clays: Preparation, properties and applications. Catal. Today.

[29] Rives V., Carriazo D., Martín C., Gil A., Korili S.A., Trujillano R., Vicente M.A. (2010). Heterogeneous Catalysis by Polyoxometalate-Intercalated Layered Double Hydroxides. Pillared Clays and Related Catalysts.

[30] Tsyganok A.I., Suzuki K., Hamakawa S., Takehira K., Hayakawa T. (2001). Mg-Al layered double hydroxide intercalated with [Ni(edta)]^2−^ chelate as a precursor for an efficient catalyst of methane reforming with carbon dioxide. Catal. Lett..

[31] Perez M.R., Pavlovic I., Barriga C., Cornejo J., Hermosin M.C., Ulibari M.A. (2006). Uptake of Cu^2+^, Cd^2+^ and Pb^2+^ on Zn-Al layered double hydroxide intercalated with edta. Appl. Clay Sci..

[32] Kaneyoshi M., Jones W. (1999). Formation of Mg-Al layered double hydroxides intercalated with nitrilotriacetate anions. J. Mater. Chem..

[33] Gutmann N.H., Spiccia L., Turney T.W. (2000). Complexation of Cu(II) and Ni(II) by nitrilotriacetate intercalated in Zn–Cr layered double hydroxides. J. Mater. Chem..

[34] Rojas R., Perez M.R., Erro E.M., Ortiz P.I., Ulibarri M.A., Giacomelli C.E. (2009). EDTA modified LDHs as Cu^2+^ scavengers: Removal kinetics and sorbent stability. J. Colloid Interface Sci..

[35] Cruz-Guzman M., Celis R., Hermosin M.C., Koskinen W.C., Nater E.A., Cornejo J. (2006). Heavy metal adsorption by montmorillonites modified with natural organic cations. Soil Sci. Soc. Am. J..

[36] Yang Z.X., Fischer H., Polder R. (2015). Laboratory investigation of the influence of two types of modified hydrotalcites on chloride ingress into cement mortar. Cem. Concr. Comp..

[37] Hongo T., Tsunashima Y., Yamasaki A. (2017). Synthesis of Ca-Al layered double hydroxide from concrete sludge and evaluation of its chromate removal ability. Sustain. Mater. Technol..

[38] AENOR Asociación Española de Normalización y Certificación, AENOR, Madrid, Spain. www.aenor.es.

[39] Mahjoubi F.Z., Khalidi A., Abdennouri M., Barka N. (2017). Zn–Al layered double hydroxides intercalated with carbonate, nitrate, chloride and sulphate ions: Synthesis, characterisation and dye removal properties. J. Taibah Univ. Sci..

[40] Reichle W.T. (1986). Synthesis of anionic clay-minerals (mixed metal-hydroxides, hydrotalcite). Solid State Ion..

[41] Crisponi G., Diaz A., Nurchi V.M., Pivetta T., Estevez M.J.T. (2002). Equilibrium study on Cd(II) and Zn(II) chelates of mercapto carboxylic acids. Polyhedron.

[42] AFNOR Association Française de Normalisation. www.afnor.org.

[43] Bayraktar A.C., Avsar E., Toroz I., Alp K., Hanedar A. (2015). Stabilization and solidification of electric arc furnace dust originating from steel industry by using low grade MgO. Arch. Environ. Prot..

[44] Farmer V.C. (1974). The Infrared Spectra of Minerals.

[45] Calvo J.L.G., Moreno M.S., Alonso M.C.A., Lopez A.H., Olmo J.G. (2013). Study of the Microstructure Evolution of Low-pH Cements Based on Ordinary Portland Cement (OPC) by Mid- and Near-Infrared Spectroscopy, and Their Influence on Corrosion of Steel Reinforcement. Materials.

[46] Nakamoto K. (1986). Infrared and Raman Spectra of Inorganic and Coordination Compounds.

[47] (1975). Joint Committee on Powder Diffraction Standards.

[48] Suetens T., Guo M., Van Acker K., Blanpain B. (2015). Formation of the ZnFe_2_O_4_ phase in an electric arc furnace off-gas treatment system. J. Hazard. Mater..

[49] Xia D.K., Pickles C.A. (1999). Caustic roasting and leaching of electric arc furnace dust. Can. Metall. Quart..

[50] Xia D.K., Pickles C.A. (2000). Microwave caustic leaching of electric arc furnace dust. Miner. Eng..

[51] Machado J., Brehm F.A., Moraes C.A.M., dos Santos C.A., Vilela A.C.F., da Cunha J.B.M. (2006). Chemical, physical, structural and morphological characterization of the electric arc furnace dust. J. Hazard. Mater..

[52] De Souza C.A.C., Machado A.T., Lima L., Cardoso R.J.C. (2010). Stabilization of Electric-Arc Furnace Dust in Concrete. Mater. Res. Ibero Am. J..

[53] De Paula L.N., Giusto L.A., Rodrigues R.C., Castilho L.R., Magalhaes F., Rosmaninho M.G., Lima D.Q. (2013). Modification and characterization of residue electric arc furnace dust (EAFD) for application in chromium (VI) reduction reactions. Quim. Nova.

[54] Van der Sloot H., Dijkstra J. Development of Horizontally Standardized Leaching Tests for Construction Materials: A Material Based or Release Based Approach? Identical Leaching Mechanisms for Different Materials. http://www.ecn.nl/docs/library/report/2004/c04060.pdf.

[55] Lasheras-Zubiate M., Navarro-Blasco I., Álvarez J.I., Fernández J.M. (2011). Interaction of carboxymethylchitosan and heavy metals in cement media. J. Hazard. Mater..

[56] Belebchouche C., Moussaceb K., Tahakourt A., Aït-Mokhtar A. (2015). Parameters controlling the release of hazardous waste (Ni^2+^, Pb^2+^ and Cr^3+^) solidified/stabilized by cement-CEM I. Mater. Struct..

[57] Cao L., Guo J., Tian J., Xu Y., Hu M., Wang M., Fan J. (2018). Preparation of Ca/Al-layered double hydroxide and the influence of their structure on early strength of cement. Constr. Build. Mater..

